# Latent Profiles Based on Combined Risk Factors for Cognitive Decline in European Older Adults: A Retrospective Study Based on the SHARE HCAP Project

**DOI:** 10.3390/neurolint17100172

**Published:** 2025-10-14

**Authors:** Johnnatas Mikael Lopes, Paola Bertuccio, Riccardo Vecchio, Giacomo Pietro Vigezzi, Lorenzo Blandi, Anna Odone

**Affiliations:** 1 Department of Public Health, Experimental and Forensic Medicine, University of Pavia, 27100 Pavia, Italy; johnnatas.lopes@univasf.edu.br (J.M.L.); paola.bertuccio@unipv.it (P.B.); riccardo.vecchio03@universitadipavia.it (R.V.);; 2 Department of Medicine, Federal University of the São Francisco Valley, Paulo Afonso 56304-917, BA, Brazil; 3 Medical Direction, IRCCS Istituti Clinici Scientifici Maugeri, 27100 Pavia, Italy; 4 School of Public Health, Vita-Salute San Raffaele University, 20132 Milan, Italy; blandi.lorenzo@hsr.it; 5 Medical Direction, Fondazione IRCCS Policlinico San Matteo, 27100 Pavia, Italy

**Keywords:** dementia, risk factors, latent class analysis, public health, prevention

## Abstract

**Background/Objectives**: Cognitive decline is common in ageing, ranging from mild to severe manifestations. Although several modifiable risk factors have been identified, they are typically examined individually. This study aimed to identify latent profiles based on combinations of dementia risk factors and to quantify the associations with cognitive impairment in a European population of older adults. **Methods**: Based on the SHARE HCAP project, we conducted a retrospective longitudinal study by linking individual data from wave 6 (2015) and wave 9 (2021–2022). The sample included 2685 individuals aged 50+. The study outcome was cognitive status, assessed using standardised neurological tests and questionnaire and categorised as normal cognition, mild cognitive impairment (MCI), or severe cognitive impairment (SCI). The exposures included clinical, psychosocial, and lifestyle variables. Latent Class Analysis (LCA) was applied to identify distinct profiles, and multinomial logistic regression models were carried out to estimate associations between latent profiles and cognitive status, expressed as odds ratios (ORs) with 95% confidence intervals (CI). **Results**: The study sample included 2326 participants, of whom 25.1% with MCI and 9.4% with SCI. Through LCA, we identified four profiles: *Low-risk*, *Combined Cluster*, *Inactive Behaviour*, and *Cardiometabolic Risk*. Compared with the *Low-risk* profile, the odds of MCI were significantly higher for *Combined Cluster* profile (OR = 3.11; 95% CI: 2.38–4.06) and CR (OR = 1.44; 95% CI: 1.07–1.93). For SCI, elevated odds were observed for *Combined Cluster* (OR = 7.30; 95% CI: 4.47–11.92), *Cardiometabolic Risk* (OR = 2.31; 95% CI: 1.31–4.05), and *Inactive Behaviour* (OR = 1.87; 95% CI: 1.01–3.48). **Conclusions**: Four latent profiles were identified, each showing distinct associations with MCI and SCI. The *Combined Cluster*—characterised by poor mental health, limited physical activity, and hypertension—showed the highest odds of cognitive impairment. Public health strategies should prioritise integrated actions against these risk factors.

## 1. Introduction

Ageing is associated with several morbidities, one of which is cognitive decline, manifesting in diverse magnitudes and dimensions [1]. The deficit in cognitive function can range from a subjective cognitive decline, through mild cognitive impairment [MCI] recognised by psychometric instruments, to severe cognitive impairment or dementia states [SCI] that incapacitate mainly elderly people [2]. Furthermore, cognitive decline cases present diverse clinical profiles, which complicates the etiologic, diagnostic and prognostic process [3].

From an etiological perspective, it is established that the milder stages of cognitive impairment serve as risk factors for deterioration [4]; however, there are underlying risk factors influencing the clinical manifestation that can modulate the onset of cognitive dysfunction, including high blood pressure, diabetes, depression, elevated low-density lipoprotein cholesterol [LDL-C], hearing and visual loss, traumatic brain injuries [TBIs], excessive alcohol consumption, smoking, obesity, sedentary lifestyle, low education, and social isolation [5]. More recently, self-perceived health, which is a global indicator of health and a predictor of future health, has been identified as a potential sign of cognitive decline [6] as well as multimorbid states limitation [7].

Given the numerous modifiable risk factors [8] and the distinct cognitive [9] and clinical profiles associated with dementia [10], an exclusive focus on the risk factors for cognitive decline as single entities presents a public health shortcoming. This limitation hinders our ability to identify subpopulations at varying levels of risk [11], as we remain unaware of the most detrimental combinations of risk factors contributing to cognitive decline [12]. Identifying profiles of risk enables the targeting of specific and economically efficient interventions for potential populations with an heterogenous distribution of risk factors [11]. However, the combination of these risk factors for SCI, generating different latent clinical profiles, has been minimally investigated [13].

Recently, some researchers have initiated approaches to evaluate the combination of factors in different profiles of cognitive decline or risk of development [13,14,15,16]. Mouchet et al. investigated, in a North American cohort, the existence of latent groups with different forms of progression in individuals with mild cognitive impairment in order to identify those with different rates of deterioration, delineating three forms: no progression, slow and rapid progression [15]. Similarly, Li et al. analysed data from the UK Bank prospectively on the existence of typical profiles for dementia based on socioeconomic criteria and lifestyle, identifying latent profiles with distinct predispositions to early and late dementia [16]. Chen et al. investigated the association of the trajectory of the multimorbid burden with later-life dementia in North American individuals and identified distinct profiles of dementia risk, which elevated the probability of developing SCI by 15% with each additional chronic disease [17].

This evidence suggests the need to investigate the existence of latent profiles of risk factors for dementia based on demographic, clinical and lifestyle data in diverse populations [11,12]. This strategy contributes to the development of a risk stratification model, which is extremely useful in the health management of chronic health conditions [18].

The aim of this research is to identify latent profiles of individuals based on combinations of risk factors for dementia, using latent class analysis in a population cohort of European elderly people. In addition, we intend to estimate the risk of MCI and SCI among the identified latent profiles, as well as stratification in cognitive domains deterioration.

## 2. Materials and Methods

### 2.1. Design and Setting

This is a retrospective longitudinal study based on data from the Survey of Health, Ageing and Retirement in Europe (SHARE) wave 6 (2015) and the first release of the SHARE Harmonised Cognitive Assessment Protocol (SHARE-HCAP 1.0) conducted during the wave 9 (2022) [19]. SHARE is a large-scale cross-national panel survey of individuals aged 50 and older, providing longitudinal individual-level data from 27 European countries and Israel [20]. SHARE HCAP, integrated within Wave 9 of the SHARE study, provides an in-depth assessment of cognitive function among a subsample of participants aged 65 years or older from five countries (Czech Republic, Denmark, France, Germany, and Italy). SHARE HCAP relies on a harmonised protocol for the assessment of cognitive functions through a standardised battery of cognitive tests [21].

Baseline exposure information was retrieved from Wave 6, while data on outcomes were retrieved from SHARE-HCAP [12]. The SHARE-HCAP dataset was linked to harmonised SHARE wave 6 data using the unique identifier variable mergeid, a 12-character code consistent across all survey waves [21].

### 2.2. Data Collection

SHARE data were collected using computer-assisted interviews covering several pieces of information, including sociodemographic, economic, lifestyle, mental and physical health-related dimensions, as well as socio-family context [20]. SHARE-HCAP included two data collection tools: (i) a battery of cognitive tests; (ii) a questionnaire administered to the participant with someone close to him/her by advanced trained interviewers, which includes variables on cognitive functions and ability to perform daily living tasks [19]. Cognitive tests provided detailed cognitive assessments across four core dimensions: orientation and visuospatial, memory, executive functioning, and language and fluency [9].

### 2.3. Study Sample

A sample of 2685 individuals from SHARE-HCAP was included (Czech *n* = 501; Danish *n* = 573, French n = 528, German *n* = 547, and Italian *n* = 537). Individuals were included in the SHARE-HCAP subsample based on the same criteria as the larger SHARE project [20] and in the response to the verbal fluency test in order to maximise the probability of selecting individuals with cognitive alterations [19]. The measured indicators presented a variation in missing data between 0.07% (MMSE Orientation) and 21.9% (WMS-IV logical memory recognition). This last measurement was collected at the end of the test battery, which may have generated greater losses due to respondent fatigue [21].

For our analysis, we excluded individuals with no information on their cognitive status in SHARE-HCAP (Figure S1). Exclusion from the SHARE-HCAP classification algorithm was based on the lack of information for the variables of education level or test battery scores, which makes standardisation and comparison with the normative sample impossible [21]. In participant wave 6, individuals with a diagnosis of dementia, Parkinson’s disease and/or a cognitive index below 1 standard deviation from the mean for educational level (less than upper secondary, upper secondary or vocational, and tertiary) and age (<60 years, 60–69 years, 70–79 years, and 80 or older) [22].

### 2.4. Study Variables

The primary outcome was the cognitive status that classified participants into three categories: normal cognition, mild cognitive impairment (MCI), and severe cognitive impairment (SCI). In accordance with the HCAP, it was obtained through a multistep statistical approach including exploratory and confirmatory factor analyses and a deterministic algorithm proposed by Börsch-Supan et al. [19]. In addition, to better explore the outcome, four different cognitive domains were considered based on the following cognitive tests: the Mini-Mental State Examination (MMSE), assessing memory, attention, orientation and calculation; the verbal fluency test (RF), for semantic memory and executive function; the Constructional Praxis test (CP) immediate, evaluating visuospatial and constructional abilities; and CP delayed, which assesses the cognitive function of planning and adaptation.

We selected a set of 15 observed binary variables, recognised as dementia risk factors [6]. These included hypertension, diabetes, and hypercholesterolemia (yes/no, based on self-reported medical diagnosis); depression, based on the EURO-D score ≥ 4; self-rated hearing, and self-rated vision (excellent/very good/good or fair/poor); obesity defined as body mass index (BMI) ≥ 30 kg/m^2^; smoking habits if participants had ever smoked daily for at least one year; alcohol abuse, defined as having consumed six or more drinks on a single occasion more than once in the past three months; sedentary lifestyle, identified if participants performed less than one vigorous exercise or two moderate exercises a week; low educational level, based on the simplified version of the International Standard Classification of Education (ISCED-97) codes (less than upper secondary education); loneliness was assessed by self-reported frequency of feelings of being left out and classified as with loneliness (some of the time/often) and without loneliness (hardly ever/never); self-perceived health was identified as adequate (excellent/very good/good) and poor perception (fair/poor), as soon as health-related limitations in daily activities, with limitation (severely limited/limited but not severely) and without limitation (not limited).

Finally, as covariates, we considered sex (male/female), age (<65, 65–74, 75–84, 85+), and area of residence (urban/rural), as derived from the wave 6.

### 2.5. Statistical Analysis

Percentages were used to describe the distribution of the selected observed variables (i.e., risk factors) and the study outcome. Differences between groups were verified through the chi-square test.

The statistical analysis consisted of two main steps. First, to classify participants into different latent classes (i.e., profiles) of possible predictors of cognitive status, we performed a multilevel latent class analysis (LCA) model on the 15 selected dementia risk factors. The number of classes were estimated based on the evaluation of the model’s quality indicators in the definition of the groups, which are: Akaike Information Criterion (AIC), Bayesian Information Criterion (BIC) and Entropy [23]. The latent class model was selected based on the lowest AIC and BIC values as well as Entropy closer to 1 [24]. The analysis was performed by creating a class up to the number of classes where there was no further reduction in the AIC and BIC values with adequate entropy. Classes with very low frequency of observations compared to the others (<15% with two classes or <10% with three or more classes) were not accepted [14]. Classes with well-distributed frequency and theoretical parsimony will be preferred when selecting the most appropriate model [23]. For the interpretation, latent profiles were characterised by item response probabilities, with values ≤ 0.20 indicating low probability and ≥0.80 high probability of endorsing each observed variables [25].

In the second step, associations between latent profiles and cognitive status (i.e., normal, MCI, and SCI) were estimated by a multinomial logistic regression model, adjusted for sex, age, and area of residence. Results were expressed as odds ratios (ORs) with 95% confidence intervals (CIs). The same model was carried out in stratified analyses in subgroups of sex, age (<65; ≥65), and area of residence (urban; rural).

Finally, we compared mean scores in the four cognitive tests in the identified latent profiles through Univariate Analysis of Variance (ANOVA) and Bonferroni post hoc. All analyses were conducted through the R^®^ software, version 4.2.2 (, the statistical package gLCA of [16] for latent class analysis, and the “survey” package for multinomial regression analysis.

## 3. Results

The SHARE HCAP sample consisted of 2685 older adults. Of these, 359 (13.4%) data were excluded due to the absence of some classification criterion, for a total of 2326.

Table 1 presents the distribution of sociodemographic and selected risk factors according to cognitive status. Overall, 25.1% (n = 583) of the participants had MCI, while 9.4% (n = 219) had SCI. The percentage of individuals with SCI was higher in Italy (8.0%), followed by the Czech Republic (6.1%); similar between men (5.2%) and women (6.5%); and higher at increasing ages (0.1% in the age group <65 and 16.6% in the 85+). Furthermore, individuals with SCI were less educated (9.5%) and reported more frequent loneliness (14.5%), multimorbidity (10.4%), hearing loss (10.3%), visual loss (13.5%), hypertension (7.6%) and depression (8.5%). Limited activities due to health conditions (8.4%) and poor self-perception of health (11.9%) also showed higher percentages of SCI.

The LCA identified a four-class model as the most interpretable and parsimonious solution (Table S1, Figure S2), based on AIC = 38,716, BIC = 39,098 and entropy = 0.593.

Table 2 displays the risk factor probabilities for the four latent classes identified by LCA. The latent profiles were named as follows: (i) *Low-risk* (30.4% of the sample), characterised by low probabilities (≤0.20–0.30) for all risk factors; (ii) *Combined cluster*, mainly characterised by multiple physical conditions, including hypertension (0.69), hearing loss (0.31), and visual loss (0.38), in combination with psychosocial and functional vulnerabilities, including depression (0.63), loneliness (0.42), sedentary lifestyle (0.78), activity limitations (0.85), and poor self-rated health (0.84); (iii) *Inactive behaviour* (23.9%), mainly characterised by sedentary lifestyle (0.70), low education (0.50), and hypertension (0.44); (iv) *Cardiometabolic risk* (15.3%), mainly characterised by hypertension (0.98), hypercholesterolemia (0.55), diabetes (0.38), smoking (0.61), along with moderate probabilities for depression (0.34), sedentary lifestyle (0.57), and limited activity (0.44). Results from stratified analyses by sex, age, and area of residence did not show any significant differences (Table S2).

Table 3 presents the ORs and 95% CIs for the associations between profiles and cognitive status, adjusted for sex, age, and area of residence. Compared to the *Low-risk* group, individuals in the *Combined Cluster* profile had significantly higher odds of both MCI (OR = 3.11; 95% CI: 2.38–4.06) and SCI (OR = 7.30; 95% CI: 4.47–11.92). Increased odds of MCI and SCI were also observed for those in the *Cardiometabolic Risk* profile (OR = 1.44; 95% CI: 1.07–1.93 and OR = 2.31; 95% CI: 1.31–4.05, respectively) and *Inactive Behaviour* profile (OR = 1.87; 95% CI: 1.01–3.48 for SCI).

**Figure 1:**
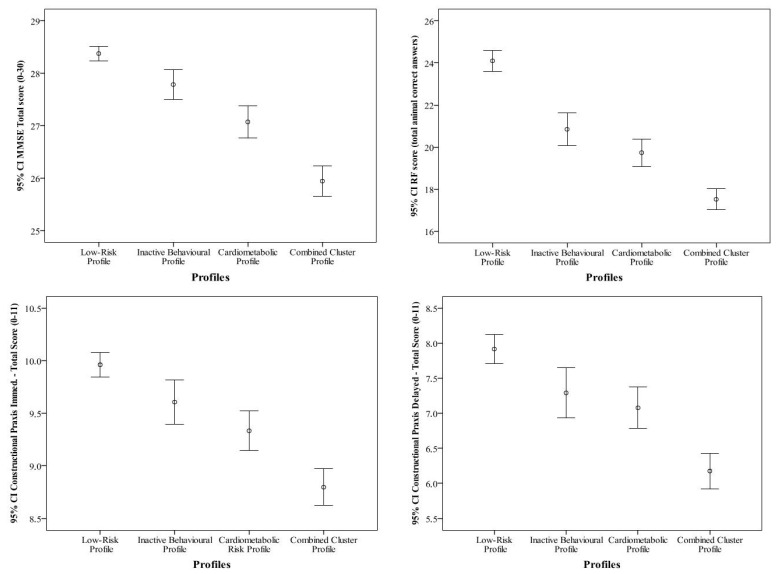
Distribution of cognitive domain scores according to the four latent profiles identified by LCA.

Figure 1 shows the mean scores and 95% CI of four cognitive domains (i.e., MMSE, RF, CD immediate, and CD delayed) according to the four latent profiles. Across all cognitive domains, individuals classified in the *Combined cluster* consistently showed the lowest cognitive scores (26.1 for MMSE, *p* < 0.001; 18.0 for RF, *p* < 0.001; 8.9 for CP immediate, *p* < 0.001; 6.2 for CP delayed, *p* < 0.001). This was also evident within each cognitive status group (normal, MCI, SCI), Figure S3. Conversely, participants belonging to the *Low-risk* profile consistently showed the highest cognitive scores (28.5 for MMSE, *p* < 0.05; 24.2 for RF, *p* < 0.001; 10.0 for CP immediate, *p* < 0.05; 7.9 for CP delayed, *p* < 0.05).

## 4. Discussion

The study confirmed the presence of underlying latent profiles based on combinations of dementia-related risk factors, each associated with different degrees of cognitive decline. The four identified profiles showed a varying risk for MCI and SCI and across cognitive domains, such as executive functioning, memory, planning, and adaptive abilities which reveals the ability to capture differences in cognitive reserve [26].

The *Low risk* profile serves as the reference category for risk stratification, comprising individuals with a low probability of presenting modifiable risk factors for dementia. However, this cluster still showed a considerable prevalence of cognitive impairment, with 18.3% of MCI and 3.9% of SCI, representing approximately 22% of the cluster and 7% of the total cognitive outcomes. These findings suggest a potential role of genetic determinants in cognitive decline within this subgroup. In line with this, Ward et al. [27] reported that genetic influences on dementia risk are more pronounced among older adults with low frailty levels, while such an effect is minimal in those with high frailty. On the other hand, Sittichokkananon et al. revealed that genetic factors interact with lifestyle-related cardiac risk factors [28]. Although genetic predisposition to dementia is widely known, its use faces problems of acceptability in genetic screening, which can be controversial for both the general population and professionals [29], requiring less costly and rapid estimation methods.

In contrast, the *Combined Cluster* profile was associated with the highest risk of severe cognitive outcomes and the worst performance across cognitive domains. This cluster was characterised by a high probability of poor health perception, functional limitations, sedentary lifestyle, depressive symptoms, and hypertension. This finding aligns with the clinical definition of multimorbidity [30,31], the co-occurrence of two or more chronic conditions, which has been linked to a 20–30% increased risk of developing dementia; each additional condition further increases the risk by approximately 15% compared to individuals with one or no chronic conditions [17]. Notably, the *Combined Cluster* also exhibited a probability below 50% for some known dementia risk factors, such as hypercholesterolaemia, hearing loss, visual loss and diabetes, even if they represent the most impactful risk factors, contributing together to almost the 20% of the prevalent attributable cases of dementia worldwide [8]. In addition, the OR for MCI and SCI development among individuals with *Combined Cluster* profile is notably higher than among those in other risk profiles. This supports our hypothesis, underlining the importance of studying the effect of combined risk factors and not only as separated entities, and may suggest the presence of other contributing clinical conditions, such as neoplasms and musculoskeletal disorders [7,32,33], that, while less traditionally emphasised, are known to affect psychological well-being and functional status, thereby contributing to the broader multimorbid burden.

The *Cardiometabolic Risk* and *Inactive Behaviour* profiles represent intermediate risk strata for the development of MCI and SCI, together accounting for approximately 45% of the sample. Given their distinct characteristics, these groups may offer valuable targets for preventive interventions. The *Inactive Behaviour* profile was mainly defined by a high likelihood of a sedentary lifestyle and, in some situations, hypertension, but showed limited probability of other elements of the metabolic syndrome. Thus, it may represent a transitional risk stratum between *Low Risk* and *Cardiometabolic Risk* profiles. Its defining risk factor (i.e., physical activity), is both modest in impact and highly modifiable, highlighting its potential for effective prevention [34]. Furthermore, evidence suggests that interventions aimed at reducing sedentary behaviour may be more impactful in this intermediate group than in individuals with more advanced risk profiles, where the potential to reverse cognitive decline is lower [35].

The *Cardiometabolic Risk* profile was primarily characterised by a high prevalence of hypertension, along with varying degrees of other components of metabolic syndrome, functional limitations, poor self-perceived health, and smoking habits. These risk factors may contribute to a transition toward the *Combined Cluster* profile, reflecting a broader multimorbid state. Preventive strategies targeting this group are more complex, as they often involve chronic and partially irreversible conditions, as well as polypharmacy [36], which may pose challenges related to access, treatment adherence, and care management [36]. The characterisation of the *Inactive Behaviour* and *Cardiometabolic Risk* profiles reveals that they may represent early stages in the progression of objective cognitive decline, initially marked by sedentary lifestyle, and subsequently by the addition of hypertension and other behavioural risk factors. Sedentary behaviour is a well-established contributor to long-term cognitive decline [37], so encouraging regular physical activity in these two groups will produce a range of positive effects in mitigating the risk of dementia [38]. These benefits are likely mediated by neuroprotective [39] and psychoactive [40,41] mechanisms, as well as by improvements in other modifiable risk factors such as diabetes, hypercholesterolemia, and functional limitations [42,43].

Conversely, the onset of hypertension exacerbates neurovascular damage and impairs the integrity of the cortical and subcortical blood–brain barrier, leading to reduced metabolic clearance and neuronal loss, promoting vascular and Alzheimer’s dementia [44], a mechanism similar to that promoted by diabetes [45]. Thus, like physical activity, hypertensive control mitigates the incidence of dementia and is also a cost-effective preventive approach for various health outcomes [44,46].

Beyond the ability to stratify risk for MCI and SCI, latent profiles proved useful in segmenting performance across all cognitive dimensions investigated, Figure S3. There is insufficient evidence to discriminate which cognitive dimensions (i.e., memory, planning, adaptation, and cognitive execution) are affected at the onset of cognitive decline, and neither can we affirm that all cognitive dimensions are affected in diagnoses of cognitive decline [47,48], which hinders early diagnosis as well as the staging of cognitive conditions [49]. The latent profiles identified here constitute an indicator that estimates a continuum of deterioration of cognitive dimensions in different cognitive statuses, revealing the potential for distinct cognitive reserves mediated by the heterogeneous prevalence of modifiable risk factors [50]. Thus, an indirect measure of cognitive reserve among population subgroups may be applicable to guide both primary preventive actions in normal individuals and therapeutic actions in those with MCI and SCI, producing cost-effective impacts [51]. This analysis revealed that risk factors are associated with each other in heterogeneous and highly complex patterns. Beyond the analysis and distribution of the population into different classes, one key point emerges: risk factors for dementia are often studied individually and comprise a percentage of preventable cases, but in reality, populations are subject to multiple joint risks, and it is necessary to study both the synergistic effects of risk factors and cross-cutting public health interventions that act on multiple risk factors.

Despite the innovative contribution of this research, namely the identification of four latent profiles reflecting a gradient of risk for MCI and SCI, as well as cognitive reserve [52], some limitations should be acknowledged. First, we were unable to include certain known risk factors, such as head trauma and air pollution exposure, in the latent class model, as this information was not available in the dataset, which may have influenced the resulting profile structure. Second, the application of this model should be tested also in population contexts with greater socio-demographic variability; for instance, in educational level, social isolation, or access to care, such as in middle- and low-income countries. Finally, the inference of transition between profiles is based on probability gradients, not on a trajectory analysis. Thus, individuals in the *Low Risk* profile could theoretically transition directly to the *Combined Cluster* without progressing through the intermediate profiles. This suggests that some profiles may represent non-linear or stagnant stages, rather than sequential stages of risk evolution.

## 5. Conclusions

This study identified four latent profiles of dementia-related risk factors within the SHARE-HCAP cohort, revealing different level of risk for both mild and severe cognitive impairment (MCI and SCI). The *Combined Cluster* profile emerged as the highest-risk group for cognitive decline, followed by *Cardiometabolic Risk* profile, a pattern consistently observed across both clinical and cognitive outcomes.

These profiles offer a valuable framework for population stratification following the risk pyramid model, supporting the prioritisation of coordinated public health actions for mental wellbeing, enhanced physical activity and cardiovascular management of older people. In addition, their potential application in clinical settings may enhance the early identification of vulnerable individuals and the development of tailored, preventive strategies aligned with individual risk profiles. In the future, additional studies, especially those combining administrative health records [53,54,55], might help corroborate these and further results in order to guide population-wide public health interventions.

## Figures and Tables

**Table 1:** Distribution of socio-demographic characteristics and selected risk factors according to cognitive status.

	Cognitive Status				
	Normal N = 1524 (65.5%)	Mild N = 583 (25.1%)	Severe N = 219 (9.4%)	Total N = 2326	*p*-Value *
Country					<0.01
Germany	364 (77.7)	114 (19.4)	25 (2.7)	503 (21.6)	
Italy	230 (68.8)	143 (23.1)	72 (7.9)	445 (19.1)	
France	307 (76.7)	97 (19.5)	39 (3.7)	443 (19.0)	
Denmark	395 (80.2)	90 (15.6)	27 (4.1)	512 (22.0)	
Czech Republic	228 (74.3)	139 (19.5)	56 (6.1)	423 (18.1)	
Sex					0.41
Men	684 (71.8)	283 (23.0)	101 (5.1)	1068 (45.9)	
Women	840 (73.8)	300 (19.7)	118 (6.4)	1258 (54.1)	
Age					0.01
<65 years	564 (74.8)	180 (23.1)	20 (0.1)	818 (39.8)	
65–74 years	612 (75.5)	229 (18.5)	78 (6.0)	1010 (41.3)	
75–84 years	234 (65.3)	131 (24.2)	81 (10.5)	485 (16.8)	
85+	29 (43.2)	11 (40.2)	13 (16.6)	57 (0.1)	
Rural area					0.71
No	903 (71.9)	332 (22.5)	119 (5.6)	1354 (63.9)	
Yes	493 (74.8)	199 (19.9)	71 (5.3)	763 (36.1)	
Education level					<0.01
<Secondary education	437 (64.7)	255 (25.8)	122 (9.5)	814 (35.0)	
≥Secondary education	1087 (79.4)	328 (17.7)	97 (2.9)	1512 (65.0)	
Loneliness					<0.01
No	1186 (75.5)	412 (21.3)	118 (3.20)	1716 (78.3)	
Yes	254 (62.3)	141 (23.0)	83 (14.54)	478 (21.7)	
Multimorbidity					<0.01
No	881 (78.5)	237 (19.2)	68 (2.3)	1186 (53.7)	
Yes	565 (64.4)	320 (25.2)	136 (10.4)	1021 (46.3)	
Hearing loss					0.01
No	1189 (74.8)	427 (20.5)	138 (4.7)	1754 (79.)	
Yes	257 (62.4)	130 (27.3)	66 (10.3)	453 (20.6)	
Vision loss					<0.01
No	1233 (76.8)	419 (19.7)	128 (3.5)	1780 (80.65)	
Yes	213 (57.5)	138 (29.0)	76 (13.5)	427 (19.35)	
Hypertension					<0.01
No	706 (73.7)	217 (23.0)	68 (3.2)	991 (47.5)	
Yes	740 (71.6)	340 (20.7)	136 (7.6)	1096 (52.5)	
Diabetes					0.50
No	1252 (73.2)	444 (21.3)	161 (5.5)	1728 (83.2)	
Yes	194 (68.7)	113 (24.1)	43 (7.2)	350 (16.8)	
Hypercholesterolemia					0.24
No	1098 (73.6)	406 (20.5)	158 (5.9)	1662 (75.3)	
Yes	348 (68.4)	151 (26.7)	46 (4.9)	545 (24.7)	
Depression					<0.01
No	906 (77.9)	312 (18.3)	94 (3.8)	1312 (59.8)	
Yes	533 (65.2)	241 (26.3)	109 (8.5)	883 (40.2)	
Psychiatric disorders					0.53
No	1370 (73.7)	524 (21.5)	194 (5.8)	2268 (96.6)	
Yes	69 (74.7)	29 (22.1)	9 (3.2)	120 (3.4)	
Obese					0.64
No	1132 (72.9)	415 (21.6)	149 (5.5)	1696 (77.6)	
Yes	303 (71.3)	134 (21.8)	50 (6.9)	487 (22.4)	
Alcohol abuse					0.68
No	1089 (72.2)	451 (22.2)	168 (5.6)	1708 (77.3)	
Yes	357 (74.5)	106 (19.3)	36 (6.2)	499 (22.6)	
Smoke habits					0.72
No	741 (71.5)	296 (22.3)	121 (6.2)	1158 (52.8)	
Yes	697 (73.5)	255 (21.3)	82 (5.2)	1034 (47.2)	
Sedentarism					0.08
No	680 (76.3)	189 (19.6)	64 (3.9)	933 (42.2)	
Yes	766 (69.8)	368 (23.1)	140 (6.9)	1274 (57.7)	
Limited activity					<0.01
No Limited	896 (77.8)	267 (18.4)	85 (3.8)	1248 (56.5)	
Limited	550 (64.8)	290 (26.7)	119 (8.5)	959 (43.5)	
Poor self-rated health					<0.01
Adequate	1077 (79.3)	323 (18.2)	81 (2.5)	1481 (65.8)	
Poor	369 (59.4)	234 (28.7)	123 (11.9)	726 (34.2)	

* The *p*-value was derived from the Chi-squared test.

**Table 2:** Risk factors probabilities (%) by four latent classes.

	Low Risk	Combined Cluster	Inactive Behaviour	Cardiometabolic Risk
Sample distribution (%)	30.4	30.3	23.9	15.3
Hypertension	0.27	0.69	0.44	0.98
Diabetes	0.03	0.24	0.07	0.38
Hypercholesterolemia	0.12	0.29	0.15	0.55
Hearing loss	0.15	0.31	0.13	0.22
Visual loss	0.05	0.38	0.19	0.13
Depression	0.31	0.63	0.30	0.34
Psychiatric disorders	0.04	0.04	0.06	0.05
Obesity	0.26	0.23	0.15	0.25
Low education	0.09	0.51	0.50	0.32
Loneliness	0.07	0.42	0.22	0.11
Sedentarism	0.29	0.78	0.70	0.57
Alcohol abuse	0.35	0.15	0.10	0.31
Smoking	0.55	0.43	0.33	0.61
Limited activity	0.23	0.85	0.14	0.44
Low self-rated health	0.07	0.84	0.09	0.24

**Table 3:** Distribution of cognitive status according to latent profiles, and associations (OR; 95% CI) estimated from adjusted multinomial logistic regression model.

	Total	Normal (Ref.)	MCI		SCI	
	N (% Col)	N (% Row)	N (% Row)	OR * (95% CI)	N (% Row)	OR * (95% CI)
Low Risk	761 (32.7)	592 (77.8)	139 (18.3)	1.00	30 (3.9)	1.00
Inactive behaviour	327 (14.1)	225 (68.8)	80 (24.5)	1.38 (0.99–1.96)	22 (6.7)	1.87 (1.01–3.48)
Cardiometabolic Risk	487 (20.9)	342 (70.2)	109 (22.4)	1.44 (1.07–1.93)	36 (7.4)	2.31 (1.31–4.05)
Combined cluster	751 (32.3)	365 (48.6)	255 (34.0)	3.11 (2.38–4.06)	131 (17.4)	7.30 (4.47–11.92)

Caption: Odds ratio (OR); Confidence interval (CI); Severe cognitive impairment (SCI); Mild cognitive impairment (MCI). * Estimated through a multinomial logistic regression model adjusted by sex, age (continuous), and area of residence (rural; urban).

## Data Availability

Data are available upon reasonable request from the first author, Johnnatas Mikael Lopes.

## Supplementary Materials

The following supporting information can be downloaded at https://www.mdpi.com/article/10.3390/neurolint17100172/s1, Table S1. Goodness of fit from latent class analysis; Table S2. Stratifies analyses of the associations between cognitive status and latent profiles, in strata of sex, age, and area of residence. Figure S1. Study design and structure for selecting participants for data analysis. Figure S2. Elbow graph with the quality indicators for selecting the number of latent classes. Figure S3. Distribution of scores for cognitive domains according to latent profile and cognitive status.

## Abbreviations

The following abbreviations are used in this manuscript:

SHARE	Survey of Health, Ageing and Retirement in Europe
HCAP	Harmonised Cognitive Assessment Protocol
MCI	Mild Cognitive Impairment
SCI	Severe Cognitive Impairment
TBI	Traumatic Brain Injury
LDL-C	Low-Density Lipoprotein Cholesterol
MMSE	Mini-Mental State Examination
RF	Verbal Fluency Test (Ruff Figural Fluency Test)
CP	Constructional Praxis Test
LCA	Latent Class Analysis
AIC	Akaike Information Criterion
BIC	Bayesian Information Criterion
OR	Odds Ratio
CI	Confidence Interval
ANOVA	Analysis of Variance
ISCED	International Standard Classification of Education
