# Supplementary material for: Latent Profiles Based on Combined Risk Factors for Cognitive Decline in European Older Adults: A Retrospective Study Based on the SHARE HCAP Project
Source: Neurol Int. 2025 Oct 14;17(10):172. doi: 10.3390/neurolint17100172 (PMC12566987; doi:10.3390/neurolint17100172)

Supplementary Materials

Tabel S1. Goodness of fit from latent class analysis.

Model	loglik	AIC	BIC	entropy	df	Gsq	*p*-value	Deviance	*p*-value
2 classes	-19519	39102	39287	0.671	2368	9613	<0.01		
3 classes	-19402	38903	39186	0.658	2351	9381	<0.01	232.7	<.001
**4 classes**	**-19292**	**38716**	**39098**	**0.593**	**2334**	**9159**	**0.05**	**221.2**	**<.001**
5 classes	-19208	38582	39062	0.608	2317	8992	<0.01	167.6	<.001
6 classes	-19147	38494	39072	0.624	2300	8870	0.15	122.2	<.001
7 classes	-19115	38464	39141	0.684	2283	8806	0.10	63.6	<.001

AIC: Akaike Information Criterion; BIC: Bayesian Unformation Criterion; df: degree of freedom; Gsq: Likelihood Ratio Chi-Square (Goodness-of-fit statistics).

Table S2. Stratifies analyses of the associations between cognitive status and latent profiles, in strata of sex, age, and area of residence.

	Inactive behaviour		Cardiometabolic Risk		Combined cluster	
	MCI vs Normal	SCI vs Normal	MCI vs Normal	SCI vs Normal	MCI vs Normal	SCI vs Normal
	OR (95% CI)	OR (95% CI)	OR (95% CI)	OR (95% CI)	OR (95% CI)	OR (95% CI)
**Sex**						
Men	1.11 (0.72-1.73)	2.09 (0.98-4.46)	0.85 (0.54-1.31)	1.54 (0.72-3.32)	2.46 (1.17-3.57)	4.91 (2.57-9.38)
Women	1.95 (1.24-3.08)	2.34 (0.95-5.78)	2.65 (1.56-4.49)	1.98 (0.66-5.91)	4.07 (2.67-6.21)	8.12 (3.59-18.37)
**Age**						
<65	1.31 (0.77-2.21)	1.18 (0.21-6.62)	1.97 (1.11-3.47)	4.24 (1.02-17.55)	3.86 (2.52-5.91)	6.99 (2.16-22.65)
≥65	1.44 (0.99-2.10)	2.45 (1.33-4.52)	1.14 (0.76-1.73)	1.59 (0.80-3.14)	2.72 (1.93-3.83)	7.15 (4.15-12.31)
**Area of residence**						
Urban	1.71 (1.17-2.48)	1.96 (0.98-3.95)	1.50 (0.99-2.27)	1.61 (0.74-3.50)	3.08 (2.21-4.30)	6.89 (3.85-12.31)
Rural	1.06 (0.63-1.79)	2.99 (1.10-8.13)	1.17 (0.66-2.04)	2.31 (0.79-6.72)	3.07 (1.97-4.79)	8.07 (3.22-20.23)

Figure S1. Study design and structure for selecting participants for data analysis.

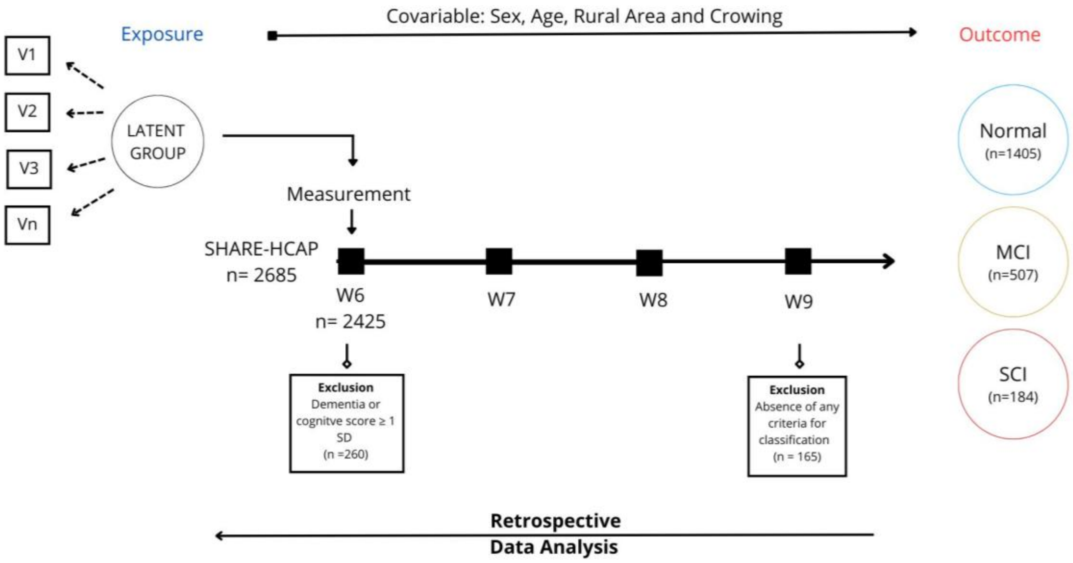

Figure S2. Elbow graph with the quality indicators for selecting the number of latent classes.

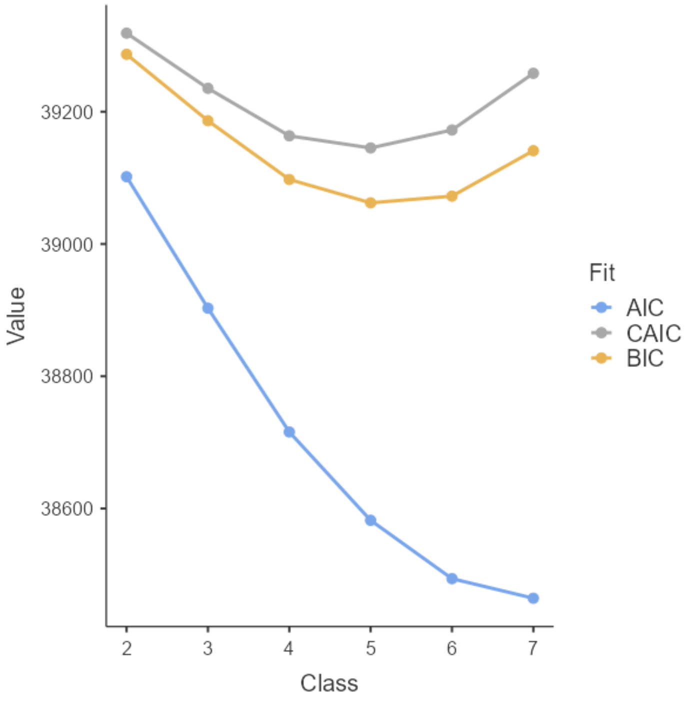

Figure S3. Distribution of scores for cognitive domains according to latent profile and cognitive status.

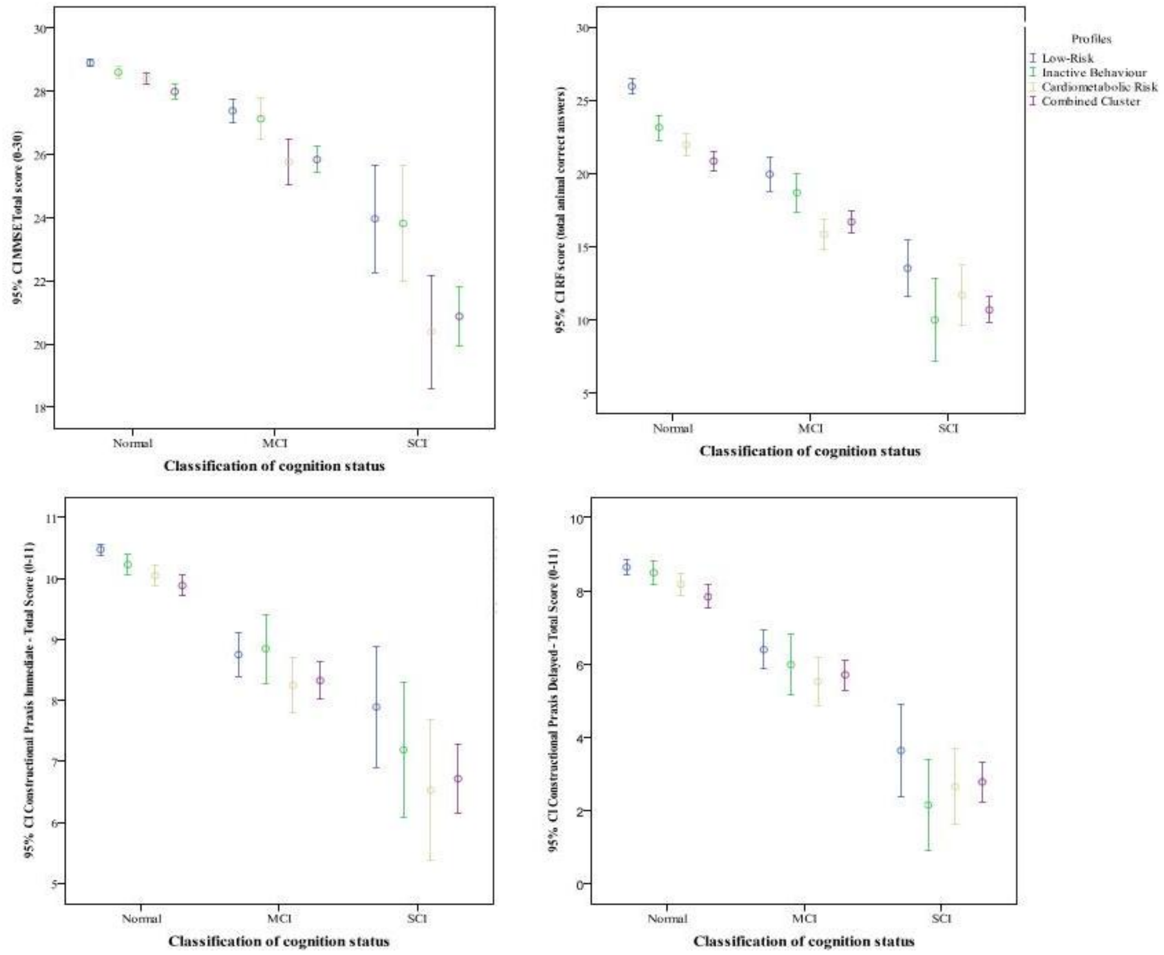

Supplement: Supplementary file 1 [file neurolint-17-00172-s001.zip › neurolint-3919679-supplementary.pdf]
